# Surgical choice for the treatment of partial intestinal ischemic necrosis caused by acute type a aortic dissection combined with malperfusion of superior mesenteric artery

**DOI:** 10.1186/s13019-024-02790-z

**Published:** 2024-05-11

**Authors:** Wenbo Yu, Yuan Liang, Jianfeng Gao, Dilin Xie, Jianxian Xiong

**Affiliations:** 1 The First Clinical Medical College of Gannan Medical University, Ganzhou, 341000 China; 2 First Affiliated Hospital of Gannan Medical University, Ganzhou, 341000 China

**Keywords:** Acute type a aortic dissection, Malperfusion syndrome, Mesenteric hypoperfusion, Intestinal necrosis, Treatment strategy

## Abstract

Acute type A aortic dissection is a severe cardiovascular disease characterized by rapid onset and high mortality. Traditionally, urgent open aortic repair is performed after admission to prevent aortic rupture and death. However, when combined with malperfusion syndrome, the low perfusion of the superior mesenteric artery can further lead to intestinal necrosis, significantly impacting the surgery’s prognosis and potentially resulting in adverse consequences, bringing. This presents great significant challenges in treatment. Based on recent domestic and international research literature, this paper reviews the mechanism, current treatment approaches, and selection of surgical methods for poor organ perfusion caused by acute type A aortic dissection. The literature review findings suggest that central aortic repair can be employed for the treatment of acute type A aortic dissection with inadequate perfusion of the superior mesenteric artery. The superior mesenteric artery can be windowed and (/or) stented, followed by delayed aortic repair. Priority should be given to revascularization of the superior mesenteric artery, followed by central aortic repair. During central aortic repair, direct blood perfusion should be performed on the distal true lumen of the superior mesenteric artery, leading to resulting in favorable therapeutic outcomes. The research results indicate that even after surgical aortic repair, intestinal ischemic necrosis may still occur. In such cases, prompt laparotomy and necessary necrotic bowel resection are crucial for saving the patient’s life.

## Introduction

Acute type A aortic dissection (ATAAD) is a life-threatening disease, with a rapidly deteriorating patient condition. The mortality rate increases by 1% within 1 h after onset, and if medical intervention is not performed in time, the mortality rate within 48 h can be as high as 30-50% [1]. Approximately 40% of patients with ATAAD experience malperfusion syndrome (MPS) [2], resulting in a total mortality rate of up to 45% [3]. Malperfusion is reported as a complication associated with ATAAD in 3.7% of cases [4]. The combination of acute aortic dissection and dissection-related MPS can lead to organ ischemic necrosis and functional failure, significantly impacting patient prognosis and potentially causing adverse consequences. Due to the lack of clear treatment strategies for malperfusion, the mortality rate of malperfusion caused by acute aortic dissection (AAD) is higher than that of AAD without malperfusion [5–7]. AAD complicated with malperfusion syndrome is defined as the loss of blood supply to important organs caused by the loss of perfusion of the branch arteries secondary to the dissection [8]. Malperfusion syndrome can affect any mesentery with inadequate blood flow, but mesenteric malperfusion (MMP) presents the greatest challenge, leading to a 3 to 4-fold increase in mortality in cases of acute type A and type B aortic dissection. The incidence of MMP has been reported to range between 66% and 100% in various studies [9]. Mesenteric ischemia malperfusion is a significant and often overlooked complication of ATAAD [10], It has been reported that the incidence of mesenteric ischemia in large multicenter registries is approximately 4–6% [11]. It significantly increases the mortality rate of ATAAD by 3–4 times. Even after complete revascularization, there is still a risk of postoperative death due to ischemia/reperfusion injury [12]. Therefore, mesenteric malperfusion deserves particular attention. Preoperative mesenteric malperfusion was associated with a mortality rate of 63.2% [13, 14]. Even with early intervention, the mortality rate for these patients remains as high as 42% [15]. Consequently, the control of malperfusion is the key to improving the outcomes of surgery for ATAAD [16]. Timely surgical relief of major organ ischemia is very important to save lives [17]. Accurate and timely diagnosis and appropriate intervention are needed before aortic repair surgery to prevent irreversible organ damage [18].

## The mechanism of organ malperfusion caused by ATAAD

Aortic branch vessel involvement is defined as the extension of the dissection to the coronary, cerebral, and splanchnic arteries on computed tomography (CT) [19], Hypoperfusion syndrome can occur in all branches from the coronary artery to the bifurcation of the abdominal aorta. Therefore, when AAD is complicated with MPS. It affects almost all branches of the aorta (including the central nervous system, coronary artery involvement, liver and kidney dysfunction, and gastrointestinal ischemic necrosis). The incidence and severity of malperfusion of each organ are different. The mechanism behind the low perfusion of branch vessels is mainly due to the compression of the true lumen by the false lumen of the branch vessel dissection. Additionally, the false lumen of the branch vessel intima is perfused with blood flow [20]. An AAD can cause mesenteric ischemia by two mechanisms: first, by occluded or narrowed SMA arteries by directly progressive dissection into the vessel, and second, by occlusion of the vessel origin by a dissection flap within the aortic lumen [21, 22], known as aortic type and branch type [23]. For cases of aortic malperfusion with dynamic obstruction, central aortic surgery or fenestration is recommended, where there is an obstruction at the origin of the vessel due to a dissecting flap within the aortic lumen. Conversely, for cases of branch-type malperfusion with static obstruction, stenting or bypass grafting is preferred [24]. In cases of static obstruction, anatomic flaps track the false lumen into branch vessels, and using self-expanding stents in the true lumen is the preferred method. For dynamic occlusion, the anatomic plane maintains branch origins, blocks flow to branch vessels, and the recommended treatment involves aortic fenestration along with deploying self-expanding stents in the true lumen [25].

## Diagnosis of ATAAD combined with malperfusion of the superior mesenteric artery

### Clinical manifestation

ATAAD is characterized by sudden tear-like pain in the chest, back, or abdomen. As the dissection progresses, the pain may spread to either the distal or proximal end. Severe pain was reported as the most common initial symptom (92.3%). The majority of patients experienced back pain (79.3%), while approximately 30.2% of patients complained of chest pain [26]. When combined with malperfusion of the superior mesenteric artery, the progression of the disease can result in intestinal ischemic necrosis. This can lead to symptoms such as progressive abdominal distension, abdominal pain, disappearance of bowel sounds, abdominal tenderness, and rebound pain.

### Imaging examinations

Computed tomography angiography (CTA) has emerged as the primary imaging modality for diagnosing AAD due to its high sensitivity and specificity. In clinical practice, CTA is the preferred method for both diagnosing and differentiating AAD. It enables visualization of the intimal flap and inwards movement of the stripping intima, which leads to the formation of the true and false lumen, as well as the presence of intramural hematoma. These criteria serve as important diagnostic indicators [27]. Structures such as the thoracic cavity and pericardial cavity should be clearly displayed, along with an evaluation of the involvement of branch vessels. After diagnosing AAD, clinicians must immediately consider the possibility of malperfusion [28]. CT is considered the gold standard for diagnosing acute mesenteric ischemia. CT images reveal excessive expansion of the intestinal wall, thickening of the intestinal wall, mesenteric edema, intestinal effusion, and increased fuzzy density of the surrounding fat gap [29]. Due to the difficulty in diagnosing mesenteric infarction and its potential fatality if delayed, laparoscopic or exploratory laparotomy may be performed for patients [30]. To overcome this challenge and even identify the early stages of ischemia, fluorescein-assisted laparoscopy can be used [31].

## Treatment status of ATAAD with organ malperfusion

Organ malperfusion in aortic dissection refers to the inadequate blood supply to organs caused by obstruction of visceral arteries, leading to organ dysfunction and MPS. This syndrome is characterized by cell death, tissue necrosis, and organ failure [32]. The impact of organ malperfusion on the outcomes of AAD surgery, both in the early and late stages, is significant. Acute occlusion of the coronary, carotid, or visceral arteries sometimes leads to irreversible organ damage after aortic surgery [11, 33, 34]. Therefore, treating ATAAD with MPS remains challenging [35]. Managing poor preoperative perfusion is a major hurdle in reducing mortality associated with surgical treatment of AAD [36]. For patients with acute type A dissection, surgical replacement of diseased vessels is the best treatment option [37, 38]. To restore the perfusion of vital organs before the progression of organ dysfunction, surgical aortic surgery is often prioritized [39]. Central aortic repair should be considered for all patients with malperfusion [40]. Poor mesenteric perfusion is a relatively serious complication, and the risk of in-hospital death is high, because it is difficult to diagnose mesenteric ischemia before necrosis changes, and when it occurs, the patient’s condition has deteriorated [41]. Diagnosis of acute mesenteric ischemia in patients with AAD may be difficult as abdominal pain is a nonspecific symptom during diagnosis [22]. The occurrence of ischemia can occur at any stage of dissection treatment. When ischemia occurs, it disrupts the oxygen supply, leading to intestinal mucosal necrosis within 3 h. If left untreated, full-thickness necrosis of the intestinal wall can occur within 6 h. Therefore, diagnosing this condition during these critical hours is crucial for successful treatment [42]. Currently, for patients with ATAAD who may have low perfusion of the superior mesenteric artery, emergency central aortic repair should be prioritized after admission, unless there is persistent or severe visceral ischemia with intestinal necrosis [43]. This approach not only saves the dissecting aneurysm from rupture but also aims to restore blood supply and pressure to the true lumen, ensuring that branch vessels can be reopened and reperfused. Postoperation, further treatment depends on the recovery of blood supply in the branch vessels and the extent of organ ischemia.

## Selection of surgical methods for ATAAD with mesenteric hypoperfusion

### Central aortic repair surgery

Aortic repair is typically performed through surgery to address malperfusion of the aortic root, coronary artery orifice, and distal end of the dissection, including brain and visceral perfusion [44]. In cases of ATAAD, central aortic repair, specifically total aortic arch replacement with stented elephant trunk implantation (Sun’s Procedure), is commonly employed to halt the progression of dissection after admission. This approach helps prevent aortic rupture, which can be life-threatening, and restore proper blood supply to branch vessels, thereby resolving the issue of poor tissue and organ perfusion. This type of surgery typically necessitates prolonged extracorporeal circulation, which can worsen the already compromised blood flow to organ tissues during the procedure. As a result, in cases where there is inadequate blood flow to the superior mesenteric artery prior to the operation, emergency central aortic repair may lead to an increased risk of malperfused intestinal necrosis post-operation. While repair of the proximal aorta often leads to the subsidence of the false lumen and improvement in tissue and organ hypoperfusion, as well as the alleviation of mesenteric ischemia symptoms, without the need for further intervention [45], there are cases where AAD involving the superior mesenteric artery can cause hypoperfusion tissue ischemia at any point before, during, or after surgery. Complete occlusion of the superior mesenteric artery can result in irreversible intestinal mucosal ischemic injury within six hours of occlusion [46]. Therefore, if central aortic repair cannot be completed within this limited time, ischemic intestinal necrosis may still occur postsurgery, significantly impacting patient prognosis and hospitalization duration. In such situations, emergency laparotomy is often necessary. If intestinal necrosis has been confirmed before surgery, central aortic repair cannot effectively improve the intestinal necrotic tissue, and exploratory laparotomy and intestinal necrosis resection are necessary.

### The superior mesenteric artery was first fenestrated and/or stented, and then the aorta was repaired by delayed opening

**Fig. 1:**
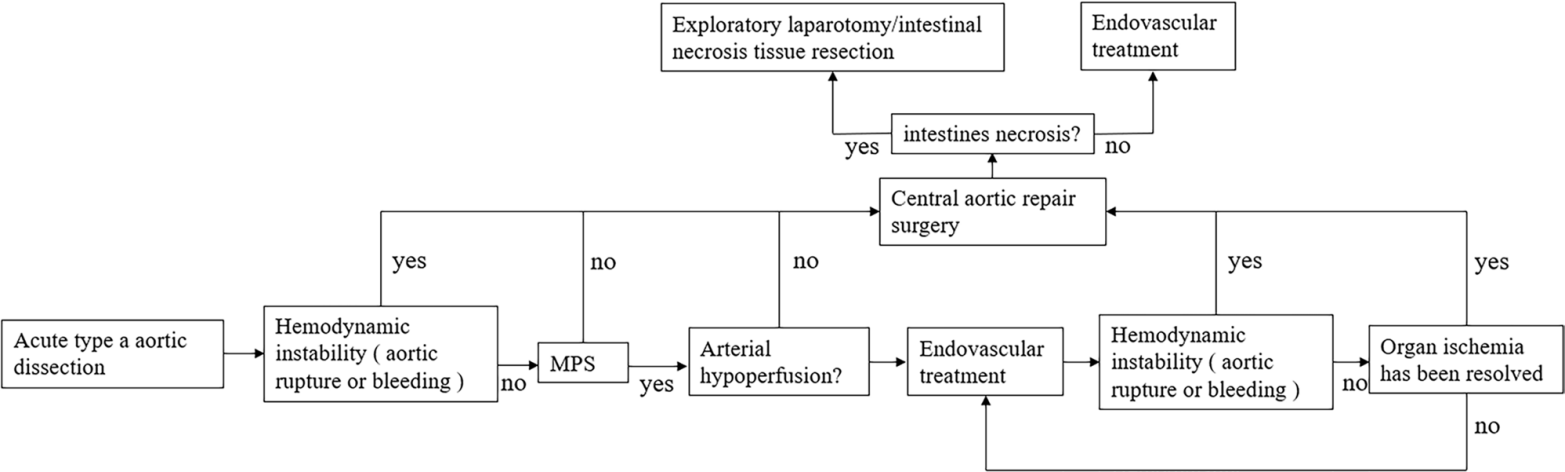
Surgical treatment options vary depending on whether the patient is hemodynamically stable or unstable

In patients with stable ATAAD and MPS, percutaneous or surgical revascularization is the initial intervention before proximal aortic repair [47, 48]. Intravascular fenestration and/or stent implantation have emerged as more favorable strategies for addressing malperfusion and delayed open aortic repair [49]. For patients diagnosed with MPS, fenestration, with or without stent implantation, can be performed prior to open surgery to repair the diseased aorta and reperfuse and stabilize the ischemic organ [50]. In 1996, the University of Michigan introduced a new treatment model for patients with ATAAD and MPS, surgical treatment options vary depending on whether the patient is hemodynamically stable or unstable (Fig. 1) [51]. This involved performing fenestration and/or stent implantation on abdominal organs experiencing severe malperfusion, followed by delayed open repair of the proximal aorta of the dissection to enable early percutaneous intravascular revascularization [52]. At Michigan Medical Center, patients with visceral and limb MPS have been treated with endovascular fenestration/stent implantation and delayed open aortic repair for 20 years, resulting in positive short-term and long-term outcomes [52–54]. Yang B.‘s study on 82 patients with acute A-type aortic dissection complicated by mesenteric hypoperfusion after endovascular fenestration/stent implantation revealed that 38% of the patients died due to organ failure or aortic rupture before undergoing open aortic repair. The remaining patients either survived through open aortic repair or were discharged without requiring it [53]. When considering central aortic repair, caution should be exercised in performing preoperative fenestration or stent implantation of the ischemic mesenteric artery. Even if the stent is implanted in the true lumen of the mesenteric artery, it remains a low-pressure cavity, and the blood supply may still not be fully restored. On the other hand, performing intima fenestration allows for blood flow to be drained from the high-pressure false lumen to the low-pressure true lumen, partially restoring blood supply. However, if central aortic repair is performed again, the pressure between the true lumen and the false lumen is reversed, and intima fenestration may lead to new hemodynamic problems due to changes in intima morphology.

### Superior mesenteric artery revascularization was given priority, and then aortic central repair was performed

Most patients with ATAAD typically undergo simple central aortic repair as the initial treatment. It is important to address mesenteric artery occlusion and intestinal ischemia through central aortic repair, which aims to restore blood supply to the true lumen and improve perfusion to branch vessels [55]. Preoperative CTA examination clearly confirmed the involvement of branch vessels. It is very important to restore true lumen blood supply as soon as possible through central aortic repair, solve the problem of low perfusion of branch vessels and reduce the time of low perfusion of tissues and organs. Research suggests that central aortic surgery or fenestration is effective for aortic malperfusion, while branch type may require stent implantation or bypass grafting [24]. A case study reported successful results in a patient with ATAAD and mesenteric ischemia who underwent a right common iliac artery to superior mesenteric artery bypass prior to aortic repair [56]. In the case reports, the patient’s intestinal necrosis was removed and the common iliac artery was bypassed to the superior mesenteric artery. Subsequently, after confirming the improvement of acidosis, ascending aortic replacement was performed on the same day, successfully saving the patient’s life [56–60]. These findings suggest that in cases where patients with ATAAD have malperfusion of the superior mesenteric artery, considering a bypass graft before central aortic repair can also be an effective treatment option. However, it is crucial to ensure stable hemodynamics and persistent mesenteric ischemia before opting for this approach.

According to Kamman et al., surgical delay in patients with malperfusion was found to be significantly associated with lower mortality. They recommend a treatment approach where branch vessel occlusion is addressed first, followed by emergency aortic repair after resolving MPS and its complications [34]. Tsagakis et al. introduced the concept of a hybrid operating room, emphasizing the revascularization of malperfusion as a priority [61]. Consequently, the strategy of prioritizing revascularization for ATAAD combined with poor mesenteric perfusion is also deemed acceptable and may yield positive outcomes [41]. The findings of these studies lean towards the conclusion that the optimal treatment strategy for emergency aortic dissection with poor mesenteric perfusion involves immediate revascularization of the superior mesenteric artery, followed by central aortic repair once the low perfusion of the superior mesenteric artery improves. The literature recognizes the catastrophic consequences of acute superior mesenteric artery occlusion and the need for emergency revascularization in patients with type A aortic dissection before central aortic repair [62].

### Direct perfusion of the distal true lumen of the superior mesenteric artery was performed while the central aorta was repaired

ATAAD can lead to various types of superior mesenteric artery hypoperfusion ischemia, each requiring different treatment measures. The aortic type involves direct compression of the superior mesenteric artery by the aortic false lumen. Remission can be achieved through simple central aortic repair in this case. On the other hand, the branch vascular type necessitates direct intervention in the superior mesenteric artery. In some patients with ATAAD, urgent central aortic repair becomes necessary due to the presence of a ruptured aorta and coronary artery ischemia after admission. In such cases, a transverse incision can be made on the superior mesenteric artery to enable direct blood perfusion. Wataru Kato et al. reported two cases of ATAAD with superior mesenteric artery ischemia, where direct blood perfusion was performed on the distal true lumen of the superior mesenteric artery during central aortic repair for the patient [63]. This approach yielded positive therapeutic outcomes and successfully avoided the need for intestinal resection.

## Conclusion and foresight

For patients with ATAAD, it is crucial to determine the blood supply of each branch before surgery, assess the level of ischemia, and choose an individualized treatment plan based on the specific conditions. In cases where there is poor blood flow to the superior mesenteric artery, central aortic repair can still be performed to effectively address the blockage caused by ATAAD and promptly restore blood supply to the branch vessels. However, if the patient experiences worsening abdominal pain postsurgery and auxiliary examinations reveal signs of intestinal necrosis, prompt consideration should be given to the possibility of intestinal ischemic necrosis. In such cases, emergency laparotomy should be performed to remove the necrotic bowel, which is an effective remedial treatment measure.

## Data Availability

Not applicable.

## Abbreviations

ATAAD: Acute type A aortic dissection
MPS: Malperfusion syndrome
AAD: Acute aortic dissection
CT: Computed tomography
CTA: Computed tomography angiography
Sun’s Procedure: Total aortic arch replacement with stented elephant trunk implantation
